# Parenting and Peer Victimization in the Development of Callous-Unemotional Behaviors: Moderation by Irritability and Basal Cortisol

**DOI:** 10.1007/s10802-025-01343-9

**Published:** 2025-06-23

**Authors:** Gretchen R. Perhamus, Jamie M. Ostrov, Dianna Murray-Close

**Affiliations:** 1 Department of Psychology, University at Buffalo, The State University of New York, Buffalo, NY USA; 2 Department of Psychological Science, The University of Vermont, Burlington, VT USA

**Keywords:** Callous-unemotional, Peer victimization, Parenting, Irritability, Cortisol

## Abstract

The present study tested three aims regarding the socializing roles of peer victimization and harsh parenting in the development of callous-unemotional (CU) behaviors. First, we investigated whether peer victimization’s promotive effects on the development of CU behaviors extend downward to early childhood and persist above effects of harsh parenting. We then considered whether, consistent with recent theoretical models, the effects of family and peer stressful experiences may be stronger for those who are emotionally (i.e., higher irritability) or physiologically (i.e., higher basal salivary cortisol) sensitive. Aims were tested over one year across the transition from preschool to kindergarten using a community sample (*N* = 263, *M*_age_ = 4.32 years, SD = 0.31 years, 47.7% female). Harsh parenting and CU behaviors were measured using parent report. Irritability and physical and relational victimization were measured using teacher report. Basal cortisol was assessed from saliva samples collected on three consecutive days in the morning. Hypothesized effects of peer victimization were not supported. However, consistent with hypotheses, harsh parenting predicted increases in CU behaviors specifically for youth with high levels of irritability (i.e., > 2.12 SDs from the mean; *B* = 0.26, *p* =.05). Finally, lower cortisol directly predicted increased CU behaviors (*B* = − 0.23, *p* <.001). Findings provide support for negative emotional reactivity as a moderating factor in the effects of harsh parenting on the development of early childhood CU behaviors, whereas HPA axis hypoactivity may directly increase risk.

**Supplementary Information:**

The online version contains supplementary material available at 10.1007/s10802-025-01343-9.

## Introduction

Callous-unemotional (CU) behaviors are characterized by a constellation of deficits in empathy, guilt, and remorse; shallow or superficial emotional expression; and disturbances in contingency learning (Frick et al., 2014; Waller & Hyde, 2017). CU behaviors are also associated with deficits in emotion (especially fear) processing and insensitivity to punishment cues, which are thought to impair rule internalization and the development of moral emotions (Wakschlag et al., 2018). CU behaviors and traits are identical in definition, but CU traits is used in later developmental periods and was initially conceptualized as a downward extension of the affective deficits seen in adult psychopathy (Frick et al., 2014; Waller & Wagner, 2019). However, in early childhood (i.e., ages 3–5), the use of the term “CU behaviors” reflects an ethical decision intended to mitigate potential iatrogenic effects of suggesting that there are young children who possess stable “traits” theoretically linked to the stigmatized construct of adult psychopathy (Waller & Hyde, 2017). Nevertheless, CU behaviors in early childhood are predictive of higher levels of CU traits, proactive aggressive behavior, and severe antisocial behavior across middle childhood and adolescence (Waller & Hyde, 2017, 2018).

The preschool years and the transition to kindergarten reflect an especially important developmental period for studying the development of CU behaviors. Cognitive and affective empathy, perspective taking, and moral emotions all show rapid growth during early childhood, and disruptions in these developmental processes may set the stage for the development of CU behaviors (Waller & Hyde, 2017, 2018). The transition to kindergarten marks a period of increased expectations from adults and peers, with problems upon school entry predicting downstream difficulties across emotional, behavioral, and social domains (Rimm-Kaufman & Pianta, 2000), and a time in which CU behaviors are malleable in response to treatment efforts (Fleming et al., 2023; Graziano et al., 2024).

### Parenting and CU Behaviors

High harshness in the caregiver-child relationship has been a key component of recent developmental models of CU behaviors. In fact, harsh parenting, characterized as highly punitive and emotionally-driven, has been established as a promotive factor in the development of CU behaviors (Hyde et al., 2016; Kimonis, 2023; Waller et al., 2013; Waller & Hyde, 2017; Waller & Wagner, 2019). For instance, observed and parent-reported harsh parenting predicted increasing CU behaviors from ages 2 to 4 in a high-risk community sample (Waller et al., 2012), and CU behaviors were positively reciprocally associated with harsh parenting among a community sample of children and their adoptive parents from 2.5 to 4.5 years old (Trentacosta et al., 2019). Furthermore, research with monozygotic twin pairs aged 6–11 years indicates that the effect of harsh parenting persists above and beyond effects of shared genetic and environmental factors (Waller et al., 2018).

Consistent with these findings, in the Sensitivity to Threat and Affiliative Reward (STAR) model (Waller & Wagner, 2019), harsh parenting is thought to promote pathways to the development of CU traits characterized by fearlessness through desensitization to threat and modeling of aggressive and fearless behavior (Waller & Wagner, 2019). Similarly, the Emotionally Sensitive Child Adverse Parenting Experiences– Allostatic (Over)Load (ESCAPE-AL) model proposes that, in addition to modeling threatening behavior, repeated and prolonged adverse parenting experiences place strain on a child’s stress and emotional response system which may ultimately exceed the child’s coping resources, leading the child to detach and develop CU behaviors over time (Kimonis, 2023). These models underscore the key role that harsh parenting may play in the development of early CU behaviors.

### Peer Victimization and CU Behaviors

In addition to harsh parenting, peer victimization may serve as an aspect of harsh treatment that plays a promotive socializing role in the development of CU behaviors. Although children’s relationships with parents and peers are interrelated, negative peer relationships have been shown to be associated with poorer socioemotional developmental outcomes above and beyond parenting and other household characteristics, including in early childhood (Goemans et al., 2023; Silver et al., 2010). For instance, in a community sample, peer rejection in kindergarten predicted increased likelihood of persistent high levels of externalizing problems across elementary school, above and beyond effects of familial relationships (Silver et al., 2010).

Similarly, peer victimization may serve as a salient and unique predictor of CU behaviors. Peer victimization involves the receipt of actions intended to hurt or harm another (i.e., aggression), perpetrated by a child not related to the victim (i.e., excludes sibling behavior), and may take multiple forms including physical and relational (Ostrov & Kamper, 2015). Physical victimization involves being the target of physical force intended to hurt or harm (e.g., hitting, kicking), whereas relational victimization is characterized by removal or threat of removal of a relationship (e.g., social exclusion, friendship withdrawal threats; Crick & Grotpeter, 1996; Ostrov & Kamper, 2015). Similarly to harsh parenting, peer victimization exposes children to interpersonal stress and threat and provides models of threatening and aggressive behavior.

Although there are some mixed findings (e.g., Fite et al., 2021; Fanti et al., 2019; Fanti & Kimonis, 2012; Joyner & Beaver, 2023), work has largely supported positive associations between peer victimization and CU traits in middle childhood and adolescence, especially when peer victimization is considered as a predictor of CU traits, rather than the reverse (e.g., Barker & Salekin, 2012; Perhamus & Ostrov, 2024; Sanchez & Cooley, 2024). For instance, a meta-analysis found individuals scoring high on peer victimization had greater odds of also scoring high on CU traits relative to non-victimized children (Zych et al., 2019). Likewise, both physical and relational peer victimization predicted a greater likelihood of belonging to classes characterized by high-stable or increasing CU traits from middle childhood to adolescence in a large population sample from the United Kingdom (Fontaine et al., 2018).

However, to our knowledge no studies have examined whether peer victimization predicts increases in CU behaviors in preschool or across the kindergarten transition. Given emerging peer competencies and the rising importance of the peer context during preschool and the transition to kindergarten (Rimm-Koffman & Pianta, 2000), as well as key developments in empathy and conscience (Waller & Hyde, 2017, 2018), peer victimization may be particularly important during this time. Therefore, the promotive effects of physical and relational victimization on the development of CU behaviors may extend downward into early childhood and persist over and above effects of harsh parenting during this developmental period.

### Moderating Role of Emotional Sensitivity

Socializing factors, such as harsh parenting and peer victimization, may be particularly impactful to the development of CU behaviors among individuals who are emotionally and biologically sensitive to their environment (Kimonis, 2023). The ESCAPE-AL model suggests that individuals who are emotionally and physiologically reactive to experiences of stress, and who are exposed to prolonged or extreme threatening or stressful experiences without appropriate support, may become “overloaded” and disengage from their environment emotionally and physiologically, reflected in CU behaviors (Kimonis, 2023). This view is consistent with the construct of a “secondary” pathway to CU behaviors, in which individuals who are initially high in negative emotionality and emotional reactivity are thought to develop callousness in response to negative social experiences and trauma (Craig et al., 2021; Kimonis, 2023; Yildirim & Derksen, 2015). In contrast, “primary” CU behaviors are thought to occur largely through biological predispositions toward unemotionality and fearlessness without substantial environmental input (Craig et al., 2021; Yildirim & Derksen, 2015). Consistent with the ESCAPE-AL model, we test indices of emotional (i.e., irritability; Beauchaine & Tackett, 2020) and stress system (i.e., basal hypothalamic-pituitary-adrenal [HPA] axis activity; Klimes-Dougan et al., 2001) sensitivity as moderators of the associations between peer victimization and harsh parenting and CU behaviors.

#### Irritability and CU Behaviors

Irritability, operationalized as a propensity to experience anger and frustration (Brotman et al., 2017; Leibenluft et al., 2024), consists of both a persistent mood component (i.e., tonic irritability) and emotional and behavioral temper outbursts (i.e., phasic irritability) and is a facet of negative emotionality (Beauchaine & Tackett, 2020; Leibenluft et al., 2024). Although often measured as a dimension of oppositional defiant disorder (ODD), irritability is also a feature of normative temperament variation, a symptom in over 20 DSM-5 diagnoses, and predictive of high levels of both concurrent comorbidity and heterotypic continuity across the spectra of internalizing and externalizing problems (Beauchaine & Tackett, 2020; Klein et al., 2021; Leibenluft et al., 2024). Therefore, within this study, and consistent with the ESCAPE-AL model (Kimonis, 2023), we focus on irritability as a transdiagnostic factor related to emotional sensitivity, rather than specifically as a dimension of ODD.

Some theoretical models posit that irritability and callousness are characteristic of two distinct phenotypes of disruptive behaviors, including in early childhood (Frick & Morris, 2004; Wakschlag et al., 2018). However, recent work has highlighted the developmental overlap among irritability and CU behaviors. For instance, among preschoolers oversampled for disruptive behavior problems, the angry/irritable mood domain of ODD (and, independently, the argumentative/defiant domain of ODD) was closely associated with the callous domain of CU behaviors (Bansal et al., 2021). Further, high levels of co-occurring irritability and CU behaviors were related to especially high levels of impairment across numerous socioemotional domains in a clinical sample of elementary-aged children (Waschbusch et al., 2020) and (with co-occurring headstrong/defiant symptoms) a community sample from Spain (Ezpeleta et al., 2022).

The ESCAPE-AL model has specifically called for work examining irritability as an emotional sensitivity factor and examining whether these effects may extend to threatening or stressful interpersonal situations outside of the caregiving environment, such as peer victimization (Kimonis, 2023). This study aims to respond to this call.

#### HPA Axis and CU Behaviors

CU behaviors may also develop in response to environmental and interpersonal stressors specifically for individuals who exhibit high HPA axis activity. The HPA axis is a component of the mammalian stress response system and is crucial for maintaining allostasis across several physiological systems (Lightman & Conway-Campbell, 2010). HPA axis activity is typically indexed using salivary assays of the hormone cortisol, and can be examined in regard to basal levels, diurnal patterns, or reactivity to stressors (Gunnar & Quevedo, 2007; Klimes-Dougan et al., 2001). Higher HPA axis activity, particularly basal levels, have been hypothesized to reflect greater biological sensitivity to environmental inputs (Boyce & Ellis, 2005). In fact studies have pointed to a role of *hyperactive* HPA axis functioning in the development of CU behaviors, especially at younger ages and when considering interactions with environmental or interpersonal stressors. For instance, among a high-poverty birth cohort sample, children with higher levels of conduct problems and CU behaviors in first grade demonstrated higher levels of basal cortisol and cortisol output during a reactivity task at 15 months old (Mills-Koonce et al., 2015). Furthermore, in the same sample, maternal harsh intrusion in infancy predicted low empathic and prosocial behaviors (a component of CU behaviors) in middle childhood specifically for children who also demonstrated high basal cortisol in infancy (Waller & Wagner, 2019). The ESCAPE-AL model (Kimonis, 2023) proposes that extreme or chronic interpersonal stress would place greater strain on the systems of individuals who are biologically sensitive to their environment; in turn, this combination of stressors and sensitivity would “overload” their psychophysiological systems, ultimately resulting in hypoactivity and the development of callousness (Kimonis, 2023; McEwen, 1998; Mills-Koonce et al., 2015).

Of note, *hypoactive* HPA axis functioning is theorized to be a genetically-mediated predisposing factor for the development of CU behaviors which may operate regardless of environmental risk (Hawes et al., 2009). HPA axis hypoactivity is thought to reflect fearlessness and sensation-seeking due to a lack of responsivity to the environment (Blair, 1999; Coren, 1999; Quay, 1965). These lower levels of fear reactivity are theorized to underlie disruptions in learning processes which would typically socialize youth away from engaging in CU behaviors (Waller & Wagner, 2019), consistent with the “primary” pathway to CU behaviors (Craig et al., 2021; Yildirim & Derksen, 2015).

### Current Study

The present study tested three complimentary aims. First (Aim 1), we examined whether the promotive effects of peer victimization on the development of CU behaviors previously demonstrated in middle childhood and adolescence extended downward to early childhood, and whether these effects persisted above established effects of harsh parenting. We expected both physical and relational victimization to predict increases in CU behaviors over the transition to kindergarten. We then considered whether, consistent with the ESCAPE-AL model (Kimonis, 2023), the effects of family and peer stressful experiences were stronger for those who were emotionally sensitive, reflected in higher irritability (Aim 2), and physiologically sensitive, reflected in higher basal salivary cortisol (Aim 3). We expected harsh parenting and physical and relational victimization to predict increases in CU behaviors across the transition to kindergarten for youth with high levels of irritability and higher basal HPA axis activity. These aims were tested across the transition from preschool to kindergarten using a community sample.

## Method

### Participants and Procedures

This study is a secondary data analysis of an ongoing short-term longitudinal study (Ostrov et al., 2023). Children (*N* = 263, *M*_age_= 4.32 years, SD = 0.31 years, 47.7% female) were recruited from preschools in a metropolitan area in the northeastern United States. Racial and ethnic diversity was consistent with that of the county from which participants were recruited (7.6% Asian or Pacific Islander, 5.7% Black or African American, 75.6% White, 9.9% more than one race, 1.1% other race/ethnicity; 3.1% Hispanic/Latine). The sample was primarily middle class, with some diversity with regard to family income and parental education (e.g., 26.0% had a household income below $100,000, 8.7% had a household income below $55,000; 6.3% of the caregivers responding did not have a 4-year college degree). In the fall, preschool teachers distributed consent forms to parents planning to send their children to kindergarten the following school year. Parents provided informed written consent for their and their children’s participation, children verbally assented to saliva collection, and teachers provided informed written consent to complete questionnaires. All procedures were approved by the University at Buffalo Institutional Review Board. Cohorts of participants were recruited annually. The present study uses data from the first four cohorts, recruited over the following years: Cohort 1 = Fall, 2019; Cohort 2 = Fall, 2020; Cohort 3 = Fall, 2021; Cohort 4 = Fall, 2022. This study uses data collected from the fall of children’s prekindergarten year (T1) and winter of kindergarten (T2).

### Measures

#### Authoritarian Parenting

Punitive or hostile parenting practices were assessed at T1 using parent report on the Authoritarian scale (8 items) of the Parenting Styles and Dimensions Questionnaires (PSDQ; Robinson et al., 2001). This scale includes non-reasoning/punitive (4 items; e.g., “I punish by taking privileges away from our child with little to no explanation”) and verbal hostility (4 items; e.g., “I yell or shout when my child misbehaves”) subscales. Items responses were scored from 1 (*Never*) to 5 (*Always*) and averaged. The scale has strong reliability in the past (e.g., Olivari et al., 2013) and demonstrated lower than convention, but adequate, internal consistency in the present study (Cronbach’s α = 0.65, McDonald’s ω = 0.63).

#### Physical and Relational Victimization

At T1, teachers completed the Preschool Peer Victimization measure– Teacher Report– Revised (PPVM-TR-R; Crick et al., 1999; Godleski et al., 2015) to assess relational (4 items; e.g., “This child gets left out of the group when someone is mad at them or wants to get back at them”) and physical (4 items; e.g., “This child gets hit, kicked, or pinched by peers”) victimization. Items were scored from 1 (*Never or almost never true*) to 5 (*Always or almost always true*) and were averaged within subscale. Subscales demonstrated adequate internal consistency in the present study (Relational victimization Cronbach’s α = 0.87, McDonald’s ω = 0.87; Physical victimization Cronbach’s α = 0.84, McDonald’s ω = 0.84).

#### Irritability

Irritability was assessed at T1 using a composite of teacher report on two questionnaires assessing children’s tendency to experience or exhibit anger and frustration– the Anger/Frustration subscale of the Child Behavior Questionnaire– Short From (CBQ-SF; Putnam & Rothbart, 2006; Rothbart et al., 2001; 6 items; e.g., “Gets quite frustrated when prevented from doing something they want to do”) and 4 items adapted from an observational method assessing behavioral displays of anger (e.g., “Uses toys or classroom materials roughly"; Hubbard et al., 2004). These items are similar to recently-developed measures capturing tonic and phasic irritability (Silver et al., 2025). CBQ-SF items were scored on a 1 (*Extremely untrue*) to 7 (*Extremely true*) scale; anger display items were scored on a 1 (*Never*) to 4 (*Almost always*) scale. Items were averaged within scale, then scales were standardized and averaged to create an irritability composite, consistent with prior work with this scale which has supported a unidimensional factor structure and convergence across parent and teacher report (Perhamus & Ostrov, 2021, 2023). In this study, both subscales demonstrated adequate internal consistency (Cronbach’s αs =.76–.87, McDonald’s ω =.75–.87) and were moderately correlated (*r* =.59, *p* <.001).

#### CU Behaviors

At T1 and T2, parents completed the Inventory of Callous Unemotional Traits– Preschool Version (ICU; Frick, 2004), which is a 24-item measure assessing callousness (e.g., “Does not care who they hurt to get what they want”), uncaring (e.g., reverse coded “Feels bad or guilty when they have done something wrong”) and unemotionality (e.g., “Does not show emotions”). Items are rated on a 0 (*Not at all true*) to 3 (*Definitely true*) scale and averaged. Internal consistency was robust (Cronbach’s αs = 0.81– 0.83, McDonald’s ωs = 0.83– 0.91).

#### Basal Salivary Cortisol

Saliva samples were collected at T1 on three consecutive days in the morning (between approximately 9:00am– 11:00am) using SalivaBio Children’s Swabs. Collection was completed at home (Cohorts 2 and 3) or school (Cohorts 1 and 4) depending on Covid-19 restrictions. Cohort was included as a covariate in all models to account for Covid-19 impacts. Swabs were held under children’s tongues for 90 seconds while they played a “food game” in which they viewed pictures of food and indicated if they liked the food with a thumbs up or down. This procedure was designed to stimulate saliva and maintain children’s attention. At school, children were assessed in a small group outside of their classrooms with one RA attending to one or two children at a time. Children refrained from eating, drinking, engaging in vigorous exercise (e.g., running), sleeping, and brushing teeth within the prior hour. Children must not have been experiencing symptoms of illness (e.g., fever) within the last 24 hours per their caregivers or teachers. Efforts were made to schedule school-based saliva collection on days that represented typical school days and after all children had been at the childcare center for at least 30 minutes. For home-based collection, caregivers were provided with training materials, detailed instructions, and completed daily check-ins with project staff. Caregivers were instructed to place samples in the back of their freezers until picked up by project staff.

Samples were stored at -27 °C in a laboratory freezer for up to 6 months and then packaged with dry ice and sent to Salimetrics SalivaLab (Carlsbad, CA) for assay. Samples were assayed using the Salimetrics Salivary Cortisol Assay Kit (Cat. No. 1-3002). Samples were thawed, vortexed, and centrifuged prior to assay. The assay involved a high sensitivity enzyme immunoassay, during which 25 µl of saliva were extracted from each sample and tested using a lower sensitivity limit of 0.007 µg/dL, a 0.012–3.00 µg/dL standard curve range, a 4.6% average intra-assay coefficient of variation (CV), and a 6% inter-assay CV. These criteria have been determined to be accurate by Salimetrics SalivaLab and exceed guidelines set by the National Institutes of Health. Samples with insufficient saliva or that returned biologically implausible values were excluded. Each sample was assayed in duplicate and values were averaged to create a daily index. Daily values were then averaged to create a time point value. Samples were moderately correlated across days (*r*s = 0.30 − 0.54, *p*s < 0.001; McDonald’s ω = 0.66).

#### Time Since Awakening

To account for diurnal changes in basal cortisol levels, the amount of time that passed between the child’s typical awakening time and cortisol collection time was included as a covariate in models examining cortisol. Parents reported on their child’s typical wake time during the week. Saliva collection time was recorded by undergraduate research assistants for school-based collection, and by parents for home-based collection, and the collection time was averaged across samples to create an average collection time for the time point. Both variables were then converted to a continuous numeric variable, and the child’s typical wake time was subtracted from the average collection time to create an index of time since awakening. As expected, this variable was moderately negatively correlated with basal cortisol values (*r* = −.31, *p* <.001), consistent with typical diurnal cortisol patterns which would be expected to decrease across the morning (Klimes-Dougan et al., 2001).

## Results

### Preliminary Analyses

Prior to primary analyses, descriptive data and bivariate correlations were examined. Outliers were winsorized to +/- 3 SDs from the mean (Kline, 2016). Skew (0.58–2.60) and kurtosis (-0.46–6.84) were within accepted ranges for normally distributed variables (Kline, 2016), but indicated slight elevations. Therefore, the maximum likelihood estimator with robust standard errors (MLR) was used for primary analyses. Descriptive statistics and bivariate correlations are presented in Table 1; descriptions of findings from bivariate correlations are available in the supplemental materials. Gender and cohort were included as covariates in all models based on their association with key study variables in preliminary analyses (see Supplemental Materials). Detailed analysis of missing data is available in the Supplemental Materials. In analyses, data were considered missing at random (MAR), and missing data was accounted for using full information maximum likelihood (FIML).

**Table 1:** Bivariate correlations and descriptive statistics

	1.	2.	3.	4.	5.	6.	7.
1. T1 Cortisol	-------						
2. T1 Auth Par	− 0.08	-------					
3. T1 Irritability	− 0.04	0.06	-------				
4. T1 Rel Vic	− 0.03	0.08	0.57^***^	-------			
5. T1 Phys Vic	− 0.02	0.01	0.49^***^	0.75^***^	-------		
6. T1 CU	− 0.06	0.18^**^	0.15^*^	0.14^+^	0.18^**^	-------	
7. T2 CU	− 0.25^**^	0.15^+^	0.12^+^	0.08	0.11	0.63^***^	------
*N*	193	223	251	249	247	219	172
*M*	0.24	1.55	-0.005	1.58	1.32	0.63	0.58
SD	0.23	0.32	0.87	0.65	0.46	0.28	0.28
Range	0.04–1.13	1.00–2.54	-1.00–2.68	1.00–3.53	1.00–2.77	0.00–1.47	0.08–1.46

Note. Auth = authoritarian, Par = parenting, Rel = relational, Phys = physical, Vic = victimization, CU = callous-unemotional, T1 = time 1, T2 = time 2. ^+^*p* <.10, *p <.05, **p <.01, ***p <.001

### Primary Analyses

Primary analyses were conducted using nested path analyses in Mplus Version 8.11 (Muthén & Muthén, 1998–2024). All variables were standardized prior to analyses. Supplemental Fig. 1 provides a visualization of nested models. First, T2 CU behaviors were regressed onto covariates (i.e., T1 CU behaviors, cohort, gender), authoritarian parenting, and physical and relational victimization. As we control for T1 CU behaviors, analyses reflect the association between predictors and changes (i.e., residualized change) in CU behaviors. Next, moderator main effects (i.e., irritability or cortisol) were added, followed by interaction terms (i.e., irritability or cortisol x physical victimization, relational victimization, and harsh parenting, respectively). Models examining the moderating effects of irritability and cortisol were run separately, and time since awakening was included as a covariate in cortisol models. Significant interactions were examined using the Johnson-Neyman technique for examining regions of significance (RoS), using online software developed by Preacher and colleagues (Preacher et al., 2006). Regression effects for all models are presented in Table 2.

**Table 2:** Effects from nested path models predicting T2 CU behaviors

	Effect Estimate	SE	*p*-value
Rel Vic	− 0.05	0.10	0.62
Phys Vic	0.02	0.11	0.87
**Auth Par**	0.01	0.07	0.87
**Irr**	0.05	0.08	0.52
Irr x Rel Vic	0.04	0.10	0.67
Irr x Phys Vic	− 0.03	0.09	0.69
**Irr x Auth Par**	**0.12**	**0.06**	**0.04**
**Cort**	**− 0.23**	**0.05**	**< 0.001**
Cort x Rel Vic	0.05	0.06	0.43
Cort x Phys Vic	0.08	0.12	0.52
Cort x Auth Par	0.06	0.08	0.45

Note. Irritability and cortisol effects were included in separate models. See manuscript for details on model building. Covariates not depicted for ease of communication. All predictors and the outcome variable were standardized prior to analyses, and interaction terms were constructed using standardized variables. Bolded values are statistically significant. Rel = relational, Phys = physical, Vic = victimization; Auth Par = authoritarian parenting; Irr = irritability; Cort = basal salivary cortisol

#### Main Effects Models

The model examining main effects of authoritarian parenting and physical and relational victimization on CU behaviors was just-identified. Contrary to predictions, none of these variables directly predicted change in CU behaviors over the transition to kindergarten.

#### Irritability Models

**Fig. 1:**
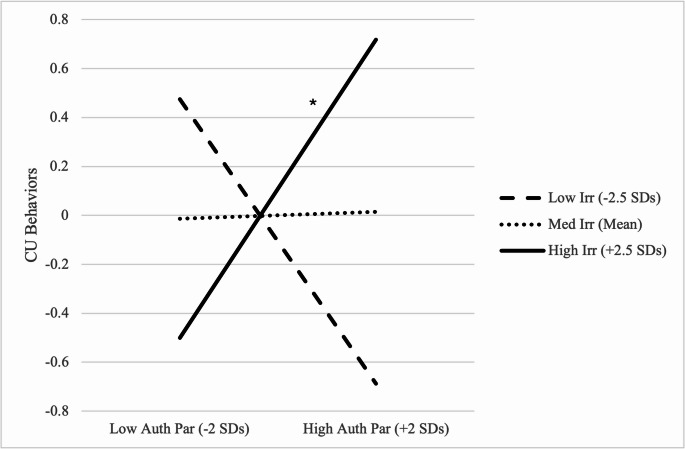
Simple slopes from authoritarian parenting x irritability predicting change in CU behaviors. Note: Simple slopes of authoritarian parenting predicting Time 2 CU behaviors with Time 1 CUbehaviors controlled. Effects are significant at levels of irritability > 2.12 SDs above the mean.The lower bound for the region of significance is outside the range of the data. CU = callous-unemotional, Irr = irritability, Auth Par = authoritarian parenting, SD = standard deviation. *p <.05

Irritability’s main effect on CU behaviors was then added to the main effects model. This model was also just-identified, and there was no significant direct effect of irritability on CU behaviors. Interaction terms between irritability and physical victimization, irritability and relational victimization, and irritability and authoritarian parenting were then added. This model was also just-identified. A significant interaction between irritability and authoritarian parenting emerged. The RoS analysis found that the effect of authoritarian parenting on CU behaviors was significant at less than − 5.74 SDs and above 2.12 SDs from the irritability mean. As the lower bound of the RoS was below the observed range of the data, only the upper bound was considered further. Specifically, at 2.12 SDs above the irritability mean, authoritarian parenting predicted increases in CU behavior over the transition to kindergarten (*B* = 0.26, SE = 0.13, *p* =.05). In other words, consistent with hypotheses, authoritarian parenting predicted increases in CU behaviors specifically at high levels of irritability. Simple slopes are depicted in Fig. 1.

#### Cortisol Models

Next, all effects of irritability were removed from the model, and the main effect of cortisol was added. Cortisol was also regressed onto time since awakening. As T2 CU behaviors and predictor variables other than cortisol were not regressed onto the time since awakening covariate, this model was over-identified and provided good fit to the data [χ^2^(5) = 5.60, *p* =.35; RMSEA = 0.02; CFI = 1.00; SRMR = 0.01]. Contrary to predictions, there was a significant negative main effect of cortisol on CU behaviors, suggesting that lower levels of cortisol predicted increases in CU behaviors across the transition to kindergarten. Finally, interaction terms were added. The model continued to provide good fit to the data [χ^2^(5) = 5.42, *p* =.37; RMSEA = 0.02; CFI = 1.00; SRMR = 0.01]. No interaction terms were significant.

## Discussion

The present study aimed to test whether physical and relational victimization predicted increases in CU behaviors across the transition to kindergarten, and if so, whether this effect persisted above and beyond prior demonstrated effects of harsh parenting (Aim 1). We then tested whether effects of harsh parenting and peer victimization were moderated by emotional and physiological sensitivity, reflected in higher irritability (Aim 2) and basal cortisol (Aim 3), respectively. The developmental period of focus represents a period of rapid change in social context and moral emotion development and remains understudied in the CU literature (Rimm-Kaufman & Pianta, 2000; Waller & Hyde, 2017, 2018). Although findings did not support the hypothesized role of peer victimization, results indicated support for the hypothesized moderating role of irritability with harsh parenting and an unexpected direct effect of cortisol.

### Effects of Peer Victimization

Despite prior work demonstrating negative impacts of preschool peer victimization on early childhood mental health (Aslan, 2018; Krygsman & Vaillancourt, 2019), and positive associations between peer victimization and CU traits in middle childhood and adolescence (Barker & Salekin, 2012; Fontaine et al., 2018; Sanchez & Cooley, 2024, Waller & Wagner, 2019; Zych et al., 2019), there were no main effects or interactions with peer victimization subtypes in the present study. Importantly, work examining the association between peer victimization and CU traits remains new, and some studies have found no association, or even negative associations, between peer victimization and CU traits (Fanti et al., 2019; Fite et al., 2021; Fanti & Kimonis, 2012; Joyner & Beaver, 2023).

Further, the ESCAPE-AL model focuses on the role of harsh parenting in the development of CU behaviors because this serves as both a source of stress and a disruption of a critical relationship which typically buffers children against the negative impacts of stress (Kimonis, 2023). In later developmental periods, such as middle childhood and adolescence, the peer context becomes more critical for serving these functions, as youth are increasingly sensitive to peer influence, spend more time with peers, rely on peer relationships for coping and support, and parental monitoring normatively decreases (Chen et al., 2015; Rubin et al., 1998). Therefore, despite being associated with poorer emotional and behavioral functioning in early childhood (Aslan, 2018; Krygsman & Vaillancourt, 2019; Ostrov, 2010), peer victimization may not serve as the type of intense stressor that disrupts coping strategies consistent with the development of CU behaviors ortraits until later in development when the peer context is more salient (Chen et al., 2015; Rubin et al., 1998). However, as the present paper is the first known study examining prospective associations between forms of peer victimization and CU behaviors in early childhood/school entry, future work is needed to further test hypotheses.

It is also worth noting that the peer context and classroom social structure change drastically during the transition to kindergarten. For instance, new peer groups are formed at school, and this change in social dynamics and social dominance hierarchies may reduce the influence of prior peer victimization (Rimm-Kaufman & Pianta, 2000). Consistent with this possibility, at the bivariate level, physical victimization was significantly correlated with CU behaviors at T1 in preschool (a nonsignificant marginal trend emerged for the correlation between relational victimization and CU behaviors), but this effect was no longer significant in kindergarten. Finally, the lack of significant predicted effects may also be a function of study limitations (e.g., impacts of the COVID-19 pandemic) as discussed below.

### Effects of Parenting and Irritability

Similarly to peer victimization, harsh parenting did not directly predict changes in CU behaviors across the kindergarten transition. This is inconsistent with prior empirical and theoretical work suggesting the importance of harsh parenting in the development of CU behaviors, including in this developmental period (Hyde et al., 2017; Kimonis, 2023; Trentacosta et al., 2019; Waller et al., 2012, 2018; Waller & Wagner, 2019). One possibility for this lack of direct effects includes the restricted range of harsh parenting observed within our sample (discussed further below). Additionally, some prior work has suggested that harsh parenting may particularly promote the development of CU behaviors when coupled with low parental warmth (Waller & Wagner, 2019), a construct which was not included in the present study.

However, consistent with hypotheses and the ESCAPE-AL model (Kimonis, 2023), harsh parenting predicted increases in CU behaviors across the kindergarten transition specifically for those demonstrating high levels of irritability. Theoretically, for those who are emotionally reactive to threat (i.e., high irritability), a harsh home environment may overload their ability to cope, resulting in emotional disengagement reflected in CU behaviors (Kimonis, 2023). This is consistent with a “secondary” pathway to the development of CU behaviors, in which children develop these characteristics in response to harsh and threatening environments and ultimately present with CU traits along with elevated negative emotionality (Craig et al., 2021; Yildirim & Derksen, 2015). Notably, prior work investigating secondary CU traits has often focused on the presence or absence of co-occurring anxiety or other internalizing problems to capture negative emotionality (Fanti & Kimonis, 2017; Fanti et al., 2013; Yildirim & Derksen, 2015), and investigations on the co-occurrence of irritability and CU behaviors remain limited. Extending evidence demonstrating the importance of considering these two constructs in tandem (Bansal et al., 2021; Barker & Salekin, 2012; Ezpeleta et al., 2022; Waschbusch et al., 2020), we provide novel evidence that irritability may represent an important facet of negative emotionality for understanding for whom harsh parenting serves as a promotive factor for CU behaviors.

### Effects of HPA Axis Functioning

Contrary to expectations and theory suggesting higher basal cortisol reflects greater sensitivity to the environment (Boyce & Ellis, 2005), salivary cortisol did not interact with either harsh parenting or peer victimization to predict change in CU behaviors across the transition to kindergarten. Although some prior studies have found positive associations between basal salivary cortisol and CU behaviors, and interactions between greater HPA axis activity and harsh parenting in predicting CU behaviors (Mills-Koonce et al., 2015; Wagner et al., 2019), these studies have often examined cortisol in infancy. It is possible that very early elevated HPA axis activity does indeed interact with harsh environments to shape the development of CU through allostatic load processes, but that this may become less evident or consistent at later ages (Mills-Koonce et al., 2015). Longitudinal research including repeated assessments of stress physiology across development are necessary to investigate this possibility (Shakiba et al., 2020).

However, cortisol was negatively directly associated with CU behaviors. Although unexpected, this finding is consistent with a large body of work suggesting that hypoactive HPA axis functioning promotes the development of CU behaviors as a genetically-mediated predisposing factor toward fearlessness and sensation-seeking, which in turn interferes with learning processes that would typically socialize children away from engaging in CU behaviors (Blair, 1999; Coren, 1999; Hawes et al., 2009; Quay, 1965; Waller & Wagner, 2019; Yildirim & Derksen, 2015). This is consistent with the primary pathway to CU behaviors hypothesized to reflect biological predispositions toward unemotionality and fearlessness and is less influenced by environmental effects (Craig et al., 2021; Yildirim & Derksen, 2015). Theoretically, youth with hypoactive HPA axis functioning, especially basal activity, may be underreactive to cues of distress, environmental stress, and punishment, which directly increases risk for the development of CU behaviors regardless of environmental context (Yildirim & Derksen, 2015).

### Limitations

Despite several strengths including a rigorous multi-method and multi-informant short-term longitudinal design and inclusion of multiple domains of functioning (i.e., biological, affective, and social), there are several key limitations that should be addressed in future research. First, there was evidence of restricted range on both CU behaviors and harsh parenting. This may in part reflect the low prevalence of CU behaviors within the general population (Wakschlag et al., 2018) and our primarily White and middle-class sample, which may be expected to show lower levels of harsh parenting (Barajas-Gonzalez & Brooks-Gunn, 2014). Parents may also have been unwilling to report that their child engages in CU behaviors or that they engage in harsh parenting, or they may have biased interpretations of their child’s behavior (Waller et al., 2013). We echo past calls for alternative informants/methods such as observers, teachers, coaches, or peers (Waller et al., 2013), and work with clinic-referred/at-risk early childhood samples.

Second, whereas the short-term longitudinal design provided a targeted examination of the key developmental transition that occurs between preschool to kindergarten, the use of only two time points and short-term nature prevented an examination of growth trajectories. Future studies are needed to assess change in CU behaviors over a longer period which extends to middle childhood, a period that is more salient for the socializing influence of peer treatment.

Third, this project was completed during the COVID-19 pandemic which added likely confounds and complications. For example, saliva was collected at home during high-precaution periods of the pandemic. Additional supports and steps were taken to ensure samples were taken at the correct time using standard procedures. However, as indicated in the supplemental materials, there was more missing data during home collection and the change in setting may have impacted the interpretation of the cortisol levels, as they may reflect differing levels of stress in school relative to home contexts. Further, those with missing saliva samples may have been those most impacted by COVID (although cortisol missingness was not associated with SES or harsh parenting in our study). Additionally, although preschools remained open throughout the majority of the pandemic in the location of data collection, kindergarten school closures and masking may have impacted the classroom context, peer relations, and teachers’ ability to report on peer victimization. Cohort differences in harsh parenting were also seen, with a post-covid cohort having lower levels than the pre-COVID cohort (see supplemental materials), suggesting the pandemic may have also influenced parenting practices.

Fourth, the assessment of basal cortisol using a single moment in time provides a limited amount of information regarding HPA axis functioning relative to more dynamic processes such as diurnal curve, the awakening response, or reactivity (Kuhlman et al., 2019; Shirtcliff et al., 2012). Although basal cortisol is somewhat stable, alternative approaches allow for a more precise estimate of the HPA axis across time and contexts. Future work will be needed to test the current hypotheses with these additional markers of HPA axis during early childhood.

Finally, internalizing symptoms (e.g., anxiety, depression) and additional aspects of externalizing symptoms (e.g., headstrong/defiant behavior, aggression) were not considered in the present study. Prior work has demonstrated unique and interactive roles of these symptoms in the development of CU behaviors (Fanti & Kimonis, 2017; Fanti et al., 2013; Yildirim & Derksen, 2015), and in interaction with parenting (Derella et al., 2019). Future work should examine the relative contributions of these additional emotional and behavioral factors, and whether the role of irritability persists above and beyond effects of these constructs.

## Conclusions & Future Directions

Overall, findings did not support the hypothesized role of peer victimization in the development of CU behaviors across the transition to kindergarten. Additional work is needed to determine the extent to which these peer experiences may contribute to the development of CU behaviors, including at later developmental stages when the peer context is especially salient (Rubin et al., 1998). However, findings are consistent with two distinct developmental pathways of CU behaviors, with one direct “primary” pathway characterized by biological predispositions toward hyposensitivity (i.e., low basal cortisol), and a “secondary” pathway characterized by temperamental hypersensitivity (i.e., irritability) which interacts with harsh environments (i.e., harsh parenting). The STAR (Waller & Wagner, 2019) and ESCAPE-AL (Kimonis, 2023) models provide new, exciting frameworks for examining underlying mechanisms in each of these pathways. An important next step is to integrate these frameworks to continue delineating how mechanisms proposed within the ESCAPE-AL model (i.e., emotional sensitivity, adverse experiences, and allostatic load) may impact sensitivity to threat and affiliative reward, as implicated within the STAR model, to shape the development of secondary CU behaviors.

These findings may also have important implications for considering different pathways to CU behavior development when designing and evaluating prevention and intervention efforts. For instance, parenting interventions focused on reducing parental harshness to address CU behaviors may be particularly impactful among youth who are emotionally reactive. This is somewhat consistent with a recent study which found that parent-child interaction therapy adapted to CU behaviors (PCIT-CU) led to steeper declines in irritable/oppositional, aggressive, and conduct behaviors among those with secondary CU behaviors relative to those with primary CU, although impacts on irritability/oppositionality deteriorated over long-term follow-up (Fleming et al., 2023). Early experiences may calibrate stress system functioning (Shakiba et al., 2020); thus, early prevention and intervention programs may also help alter stress system functioning with downstream effects for primary CU behaviors.

However, there remains a dearth of work examining intervention effects on different pathways to early CU behaviors, particularly considering underlying mechanisms and potential protective factors. For instance, interventions for CU behaviors often focus on increasing parental warmth in addition to decreasing harshness (Fleming et al., 2023; Waller & Wagner, 2019), but it is unknown whether parental warmth is a protective factor in the development of CU behaviors particularly for emotionally sensitive youth. It is further unknown whether peer relationships characterized by high levels of warmth and supportiveness (e.g., high levels of received prosocial behavior) may play a parallel protective role for emotionally sensitivity youth. Finally, it could be informative to consider the role of peer victimization along with emotional sensitivity factors when examining intervention efforts among older children, which has sometimes failed to find expected CU variant differences in outcomes (Thøgersen et al., 2022). Additional work applying a developmentally informed approach to consider the interplay among affect, psychophysiology, and social factors in the shaping of CU behaviors is needed to inform these important prevention and intervention efforts.

## Electronic Supplementary Material

Below is the link to the electronic supplementary material.

Supplementary Material 1

## Data Availability

Data and supporting information is available from the authors upon reasonable request and will be deposited in a repository following completion of the parent study.

## References

[CR1] Aslan, Ö. M. (2018). Peer rejection and internalizing behavior: The mediating role of peer victimization in preschool. *The Journal of Genetic Psychology*, *179*(4), 198–206. 10.1080/00221325.2018.1468993 29791277 10.1080/00221325.2018.1468993

[CR4] Bansal, P. S., Goh, P. K., Eng, A. G., Elkins, A. R., Thaxton, M., Smith, T. E., & Martel, M. M. (2021). Identifying the inter-domain relations among ODD, CD, and CU traits in preschool children using network analysis. *Research on Child and Adolescent Psychopathology*, *49*(10), 1289–1301. 10.1007/s10802-021-00836-7 34128173 10.1007/s10802-021-00836-7

[CR2] Barajas-Gonzalez, R. G., & Brooks-Gunn, J. (2014). Income, neighborhood stressors, and harsh parenting: Test of moderation by ethnicity, age, and gender. *Journal of Family Psychology*, *28*(6), 855–866. 10.1037/a0038242 25383794 10.1037/a0038242

[CR3] Barker, E. D., & Salekin, R. T. (2012). Irritable oppositional defiance and callous unemotional traits: Is the association partially explained by peer victimization? *Journal of Child Psychology and Psychiatry*, *53*(11), 1167–1175. 10.1111/j.1469-7610.2012.02579.x 22783837 10.1111/j.1469-7610.2012.02579.x PMC3619049

[CR5] Beauchaine, T. P., & Tackett, J. L. (2020). Irritability as a transdiagnostic vulnerability trait: Current issues and future directions. *Behavior Therapy*, *51*(2), 350–364. 10.1016/j.beth.2019.10.009 32138943 10.1016/j.beth.2019.10.009

[CR6] Blair, R. J. R. (1999). Responsiveness to distress cues in the child with psychopathic tendencies. *Personality and Individual Differences*, *27*(1), 135–145.

[CR7] Boyce, W. T., & Ellis, B. J. (2005). Biological sensitivity to context: I. An evolutionary-developmental theory of the origins and functions of stress reactivity. *Development and Psychopathology*, *17*(2), 271–301. 10.1017/s0954579405050145 16761546 10.1017/s0954579405050145

[CR8] Brotman, M. A., Kircanski, K., Stringaris, A., Pine, D. S., & Leibenluft, E. (2017). Irritability in youths: A translational model. *The American Journal of Psychiatry*, *174*(6), 520–532. 10.1176/appi.ajp.2016.16070839 28103715 10.1176/appi.ajp.2016.16070839 PMC13335380

[CR9] Chen, D., Drabick, D. A., & Burgers, D. E. (2015). A developmental perspective on peer rejection, deviant peer affiliation, and conduct problems among youth. *Child Psychiatry and Human Development*, *46*(6), 823–838. 10.1007/s10578-014-0522-y 25410430 10.1007/s10578-014-0522-y PMC4440840

[CR10] Coren, S. (1999). Arousal predisposition as a predictor of antisocial and delinquent behavior. *Personality and Individual Differences*, *27*(5), 815–820.

[CR11] Craig, S. G., Goulter, N., & Moretti, M. M. (2021). A systematic review of primary and secondary callous-unemotional traits and psychopathy variants in youth. *Clinical Child and Family Psychology Review*, *24*(1), 65–91. 10.1007/s10567-020-00329-x 33079293 10.1007/s10567-020-00329-x

[CR13] Crick, N. R., & Grotpeter, J. K. (1996). Children’s treatment by peers: Victims of relational and overt aggression. *Development and Psychopathology*, *8*(2), 367–380. 10.1017/S0954579400007148

[CR12] Crick, N. R., Casas, J. F., & Ku, H. (1999). Relational and physical forms of peer victimization in preschool. *Developmental Psychology*, *35*, 376–385. 10.1037/0012-1649.35.2.376 10082008 10.1037//0012-1649.35.2.376

[CR14] Derella, O. J., Burke, J. D., Stepp, S. D., & Hipwell, A. E. (2019). Reciprocity in undesirable parent-child behavior? Verbal aggression, corporal punishment, and girls’ oppositional defiant symptoms. *Journal of Clinical Child and Adolescent Psychology*, *49*(6), 420–433. 10.1080/15374416.2019.1603109 31059308 10.1080/15374416.2019.1603109 PMC6832786

[CR15] Ezpeleta, L., Penelo, E., Navarro, J. B., de la Osa, N., & Trepat, E. (2022). Co-developmental trajectories of defiant/headstrong, irritability, and prosocial emotions from preschool age to early adolescence. *Child Psychiatry & Human Development*, *53*, 908–918. 10.1007/s10578-021-01180-z 33939109 10.1007/s10578-021-01180-z

[CR17] Fanti, K. A., & Kimonis, E. R. (2012). Bullying and victimization: The role of conduct problems and psychopathic traits. *Journal of Research on Adolescence*, *22*(4), 617–631. 10.1111/j.1532-7795.2012.00809.x

[CR18] Fanti, K., & Kimonis, E. (2017). Heterogeneity in externalizing problems at age 3: Association with age 15 biological and environmental outcomes. *Developmental Psychology*, *53*. 10.1037/dev0000317 10.1037/dev0000317 28406655

[CR16] Fanti, K. A., Demetriou, C. A., & Kimonis, E. R. (2013). Variants of callous-unemotional conduct problems in a community sample of adolescents. *Journal of Youth and Adolescence*, *42*(7), 964–979. 10.1007/s10964-013-9958-9 23644815 10.1007/s10964-013-9958-9

[CR19] Fanti, K. A., Kokkinos, C. M., Voulgaridou, I., & Hadjicharalambous, M. (2019). Investigating the association between callous-unemotional traits with relational bullying and victimization: A cross‐national study. *Social Development*, *28*(4), 854–872. 10.1111/sode.12381

[CR20] Fite, P. J., Williford, A., Griffith, R. L., & Parker, K. (2021). Peer victimization among detained youth: The impact of callous-unemotional traits. *Child & Youth Care Forum*, *50*(4), 569–585. 10.1007/s10566-020-09593-y

[CR21] Fleming, G. E., Neo, B., Kaouar, S., & Kimonis, E. R. (2023). Treatment outcomes of children with primary versus secondary callous-unemotional traits. *Research on Child and Adolescent Psychopathology*, *51*(11), 1581–1594. 10.1007/s10802-023-01112-6 37552366 10.1007/s10802-023-01112-6 PMC10627936

[CR22] Fontaine, N. M. G., Hanscombe, K. B., Berg, M. T., McCrory, E. J., & Viding, E. (2018). Trajectories of callous-unemotional traits in childhood predict different forms of peer victimization in adolescence. *Journal of Clinical Child & Adolescent Psychology*, *47*(3), 458–466. 10.1080/15374416.2015.1105139 26890671 10.1080/15374416.2015.1105139 PMC6444182

[CR23] Frick, P. J. (2004). *The inventory of Callous-Unemotional traits (Unpublished rating scale)*. University of New Orleans.

[CR24] Frick, P. J., & Morris, A. S. (2004). Temperament and developmental pathways to conduct problems. *Journal of Clinical Child & Adolescent Psychology*, *33*(1), 54–68. 10.1207/S15374424JCCP3301_6 15028541 10.1207/S15374424JCCP3301_6

[CR25] Frick, P. J., Ray, J. V., Thornton, L. C., & Kahn, R. E. (2014). Annual research review: A developmental psychopathology approach to Understanding callous-unemotional traits in children and adolescents with serious conduct problems. *Journal of Child Psychology and Psychiatry*, *55*(6), 532–548. 10.1111/jcpp.12152 24117854 10.1111/jcpp.12152

[CR27] Godleski, S. A., Kamper, K. E., Ostrov, J. M., Hart, E. J., & Blakely-McClure, S. J. (2015). Peer victimization and peer rejection during early childhood. Journal of Clinical Child and Adolescent Psychology, 44, 380-392. 10.1080/15374416.2014.940622 10.1080/15374416.2014.940622 PMC4333131 25133659

[CR29] Goemans, A., Viding, E., & McCrory, E. (2023). Child maltreatment, peer victimization, and mental health: Neurocognitive perspectives on the cycle of victimization. *Trauma Violence & Abuse*, *24*(2), 530–548. 10.1177/15248380211036393 10.1177/15248380211036393 PMC10009486 34355601

[CR76] Graziano, P. A., Hernandez, M. L., & Dick, A. S. (2024). Examining change in callous-unemotional behaviors in young children withAttention-Deficit/Hyperactivity Disorder (ADHD) and comorbid cisruptive behavior disorders:Impact of the Summer Treatment Program for Pre-Kindergarteners (STP-PreK). *Evidence-BasedPractice in Child and Adolescent Mental Health*, 1–17. 10.1080/23794925.2024.2400877 10.1080/23794925.2024.2400877 PMC12333567 40785970

[CR30] Gunnar, M., & Quevedo, K. (2007). The neurobiology of stress and development. *Annual Review of Psychology*, *58*, 145–173. 10.1146/annurev.psych.58.110405.085605 16903808 10.1146/annurev.psych.58.110405.085605

[CR31] Hawes, D. J., Brennan, J., & Dadds, M. R. (2009). Cortisol, callous-unemotional traits, and pathways to antisocial behavior. *Current Opinion in Psychiatry*, *22*(4), 357–362. 10.1097/YCO.0b013e32832bfa6d 19455037 10.1097/YCO.0b013e32832bfa6d

[CR32] Hubbard, J. A., Parker, E. H., Ramsden, S. R., Flanagan, K. D., Relyea, N., Dearing, K. F., Smithmyer, C. M., Simons, R. F., & Hyde, C. T. (2004). The relations among observational, physiological, and self-report measures of children’s anger. *Social Development*, *13*(1), 14–39. 10.1111/j.1467-9507.2004.00255.x 10.1111/1467-8624.00460 12146736

[CR33] Hyde, L. W., Waller, R., Trentacosta, C. J., Shaw, D. S., Neiderhiser, J. M., Ganiban, J. M., Reiss, D., & Leve, L. D. (2016). Heritable and nonheritable pathways to early callous-unemotional behaviors. *The American Journal of Psychiatry*, *173*(9), 903–910. 10.1176/appi.ajp.2016.15111381 27056607 10.1176/appi.ajp.2016.15111381 PMC5008992

[CR34] Joyner, B., & Beaver, K. M. (2023). Examining the potential association between callous-unemotional traits and victimization: A behavioral genetic analysis. *Journal of Developmental and Life-Course Criminology*, *9*(3), 507–530. 10.1007/s40865-023-00228-z

[CR35] Kimonis, E. R. (2023). The emotionally sensitive Child-Adverse parenting Experiences-Allostatic (Over)Load (ESCAPE-AL) model for the development of secondary psychopathic traits. *Clinical Child and Family Psychology Review*, *26*(4), 1097–1114. 10.1007/s10567-023-00455-2 37735279 10.1007/s10567-023-00455-2 PMC10640461

[CR37] Klein, D. N., Dougherty, L. R., Kessel, E. M., Silver, J., & Carlson, G. A. (2021). A transdiagnostic perspective on youth irritability. *Current Directions in Psychological Science*, *30*(5), 437–443. 10.1177/09637214211035101 35046617 10.1177/09637214211035101 PMC8765598

[CR36] Klimes-Dougan, B., Hastings, P. D., Granger, D. A., Usher, B. A., & Zahn-Waxler, C. (2001). Adrenocortical activity in at-risk and normally developing adolescents: Individual differences in salivary cortisol basal levels, diurnal variation, and responses to social challenges. *Development and Psychopathology*, *13*(3), 695–719. 10.1017/S0954579401003157 11523855 10.1017/s0954579401003157

[CR38] Kline, R. B. (2016). *Principles and practice of structural equation modeling* (4th ed.). Guilford.

[CR39] Krygsman, A., & Vaillancourt, T. (2019). Peer victimization, aggression, and depression symptoms in preschoolers. *Early Childhood Research Quarterly*, *47*, 62–73. 10.1016/j.ecresq.2018.09.006

[CR74] Kuhlman, K. R., Robles, T. F., Dickenson, L., Reynolds, B., & Repetti, R. L. (2019). Stability of diurnal cortisol measures across days, weeks, and years across middle childhood and early adolescence: Exploring the role of age, pubertal development, and sex. *Psychoneuroendocrinology, 100*, 67-74. 10.1016/j.psyneuen.2018.09.33 10.1016/j.psyneuen.2018.09.033 PMC6333497 30292961

[CR40] Leibenluft, E., Allen, L., Althoff, R., Brotman, M., Burke, J., Carlson, G., Dickstein, D., Dougherty, L., Evans, S., Kircanski, K., Klein, D., Malone, E., Mazefsky, C., Nigg, J., Perlman, S., Pine, D., Roy, A., Salum, G., Shakeshaft, A., & Stringaris, A. (2024). Irritability in youths: A critical integrative review. *The American Journal of Psychiatry*, *181*(4), 275–290. 10.1176/appi.ajp.20230256 38419494 10.1176/appi.ajp.20230256 PMC12010774

[CR41] Lightman, S. L., & Conway-Campbell, B. L. (2010). The crucial role of pulsatile activity of the HPA axis for continuous dynamic equilibration. *Nature Reviews Neuroscience*, *11*(10), 710–718. 10.1038/nrn2914 20842176 10.1038/nrn2914

[CR42] McEwen, B. S. (1998). Stress, adaptation, and disease: Allostasis and allostatic load. *Annals of the New York Academy of Sciences*, *840*(1), 33–44. 10.1111/j.1749-6632.1998.tb09546.x 9629234 10.1111/j.1749-6632.1998.tb09546.x

[CR43] Mills-Koonce, W. R., Wagner, N. J., Willoughby, M. T., Stifter, C., Blair, C., Granger, D. A., & Family Life Project Key Investigators. (2015). Greater fear reactivity and psychophysiological hyperactivity among infants with later conduct problems and callous-unemotional traits. *Journal of Child Psychology and Psychiatry*, *56*(2), 147–154. 10.1111/jcpp.12289 24992385 10.1111/jcpp.12289 PMC4282840

[CR44] Olivari, G. M., Tagliabue, S., & Confalonieri, E. (2013). Parenting style and dimensions questionnaire: A review of reliability and validity. *Marriage and Family Review*, *49*(6), 465–490. 10.1080/01494929.2013.770812

[CR45] Ostrov, J. M. (2010). Prospective associations between peer victimization and aggression. *Child Development*, *81*(6), 1670–1677. 10.1111/j.1467-8624.2010.01501.x 21077855 10.1111/j.1467-8624.2010.01501.x

[CR46] Ostrov, J. M., & Kamper, K. E. (2015). Future directions for research on the development of relational and physical peer victimization. *Journal of Clinical Child & Adolescent Psychology*, *44*(3), 509–519. 10.1080/15374416.2015.1012723 25751392 10.1080/15374416.2015.1012723

[CR47] Ostrov, J. M., Murray-Close, D., Perry, K. J., Perhamus, G. R., Memba, G. V., Rice, D. R., & Nowalis, S. (2023). Parenting and adjustment problems among preschoolers during COVID-19. *Journal of Child and Family Studies*, *32*(1), 93–109. 10.1007/s10826-022-02439-2 36157198 10.1007/s10826-022-02439-2 PMC9488881

[CR48] Perhamus, G. R., & Ostrov, J. M. (2021). Emotions and cognitions in early childhood aggression: The role of irritability and hostile attribution biases. *Research on Child and Adolescent Psychopathology*, *49*(1), 63–75. 10.1007/s10802-020-00707-7 32975688 10.1007/s10802-020-00707-7

[CR49] Perhamus, G. R., & Ostrov, J. M. (2023). Inhibitory control in early childhood aggression subtypes: Mediation by irritability. *Child Psychiatry and Human Development*, *54*(2), 366–378. 10.1007/s10578-021-01254-y 34550506 10.1007/s10578-021-01254-y

[CR50] Perhamus, G. R., & Ostrov, J. M. (2024). Peer socialization processes in the development of callous-unemotional traits. *Development and Psychopathology*, 1–18. 10.1017/S0954579424001846 10.1017/S0954579424001846 PMC12149337 39655688

[CR51] Preacher, K. J., Curran, P. J., & Bauer, D. J. (2006). Computational tools for probing interaction effects in multiple linear regression, multilevel modeling, and latent curve analysis. *Journal of Educational and Behavioral Statistics*, *31*, 437–448.

[CR52] Putnam, S. P., & Rothbart, M. K. (2006). Development of short and very short forms of the children’s behavior questionnaire. *Journal of Personality Assessments*, *87*(1), 102–112. 10.1207/s15327752jpa8701_09 10.1207/s15327752jpa8701_09 16856791

[CR53] Quay, H. C. (1965). Psychopathic personality as pathological stimulation-seeking. *American Journal of Psychiatry*, *122*(2), 180–183. 14313433 10.1176/ajp.122.2.180

[CR54] Rimm-Koffman, S. E., & Pianta, R. C. (2000). An ecological perspective on the transition to kindergarten: A theoretical framework to guide empirical research. *Journal of Applied Developmental Psychology*, *21*(5). 10.1016/S0193-3973(00)00051-4

[CR55] Robinson, C. C., Mandleco, B., Olsen, S. F., & Hart, C. H. (2001). The parenting styles and dimensions questionnaire (PSDQ). *Handbook of family measurement techniques*, *3*(319–321).

[CR56] Rothbart, M. K., Ahadi, S. A., Hershey, K. L., & Fisher, P. (2001). Investigations of temperament at 3–7 years: The children’s behavior questionnaire. *Child Development*, *72*, 1394–1408. 10.1111/1467-8624.00355 11699677 10.1111/1467-8624.00355

[CR57] Rubin, K. H., Bukowski, W., & Parker, J. G. (1998). Peer interactions, relationships, and groups. In W. Damon (Ed.), *Handbook of child psychology* (5th ed., pp. 619–700). Wiley.

[CR58] Sanchez, C. R., & Cooley, L., J (2024). Peer victimization and callous-unemotional traits: The impact of parents and teachers. *Research on Child and Adolescent Psychopathology*, *52*(10), 1551–1564. 10.1007/s10802-024-01213-w 38819578 10.1007/s10802-024-01213-w

[CR59] Shakiba, N., Ellis, B. J., Bush, N. R., & Boyce, W. T. (2020). Biological sensitivity to context: A test of the hypothesized U-shaped relation between early adversity and stress responsivity. *Development and Psychopathology*, *32*(2), 641–660. 10.1017/S0954579419000518 31347484 10.1017/S0954579419000518

[CR61] Silver, R. B., Measelle, J. R., Armstrong, J. M., & Essex, M. J. (2010). The impact of parents, child care providers, teachers, and peers on early externalizing trajectories. *Journal of School Psychology*, *48*(6), 555–583. 10.1016/j.jsp.2010.08.003 21094398 10.1016/j.jsp.2010.08.003 PMC3017381

[CR60] Silver, J., Carlson, G. A., Farquharson, W. H., Atlas, J., & Klein, D. N. (2025). Development and validation of a multi-informant scale for assessing youth tonic and phasic irritability. *Research on Child and Adolescent Psychopathology*, 1-16. 10.1007/s10802-025-01318-w10.1007/s10802-025-01318-w40232566

[CR75] Shirtcliff, E. A., Allison, A. L., Armstrong, J. M., Slattery, M. J., Kalin, N. H., & Essex, M. J. (2012). Longitudinal stability and developmental properties of salivary cortisol levels and circadian rhythms from childhood to adolescence. *Developmental Psychobiology*, *54*(5), 493-502. 10.1002/dev.20607 10.1002/dev.20607 PMC3270212 21953537

[CR62] Thøgersen, D. M., Elmose, M., Viding, E., McCrory, E., & Bjørnebekk, G. (2022). Behavioral improvements but limited change in callous-unemotional traits in adolescents treated for conduct problems. *Journal of Child and Family Studies*, *31*(12), 3342–3358. 10.1007/s10826-022-02435-6

[CR63] Trentacosta, C. J., Waller, R., Neiderhiser, J. M., Shaw, D. S., Natsuaki, M. N., Ganiban, J. M., Reiss, D., Leve, L. D., & Hyde, L. W. (2019). Callous-unemotional behaviors and harsh parenting: Reciprocal associations across early childhood and moderation by inherited risk. *Journal of Abnormal Child Psychology*, *47*(5), 811–823. 10.1007/s10802-018-0482-y 30306411 10.1007/s10802-018-0482-y PMC6459732

[CR64] Wakschlag, L. S., Perlman, S. B., Blair, R. J., Leibenluft, E., Briggs-Gowan, M. J., & Pine, D. S. (2018). The neurodevelopmental basis of early childhood disruptive behavior: Irritable and callous phenotypes as exemplars. *The American Journal of Psychiatry*, *175*(2), 114–130. 10.1176/appi.ajp.2017.17010045 29145753 10.1176/appi.ajp.2017.17010045 PMC6075952

[CR67] Waller, R., & Hyde, L. W. (2017). Callous–unemotional behaviors in early childhood: Measurement, meaning, and the influence of parenting. *Child Development Perspectives*, *11*(2), 120–126. 10.1111/cdep.12222 28824706 10.1111/cdep.12222 PMC5560612

[CR68] Waller, R., & Hyde, L. W. (2018). Callous-unemotional behaviors in early childhood: The development of empathy and prosociality gone awry. *Current Opinion in Psychology*, *20*, 11–16. 10.1016/j.copsyc.2017.07.037 28822897 10.1016/j.copsyc.2017.07.037 PMC5965673

[CR70] Waller, R., & Wagner, N. (2019). The sensitivity to threat and affiliative reward (STAR) model and the development of callous-unemotional traits. *Neuroscience & Biobehavioral Reviews*, *107*, 656–671. 10.1016/j.neubiorev.2019.10.005 31618611 10.1016/j.neubiorev.2019.10.005

[CR66] Waller, R., Gardner, F., Hyde, L. W., Shaw, D. S., Dishion, T. J., & Wilson, M. N. (2012). Do harsh and positive parenting predict parent reports of deceitful-callous behavior in early childhood? *Journal of Child Psychology and Psychiatry and Allied Disciplines*, *53*(9), 946–953. 10.1111/j.1469-7610.2012.02550.x 22490064 10.1111/j.1469-7610.2012.02550.x PMC3454481

[CR65] Waller, R., Gardner, F., & Hyde, L. W. (2013). What are the associations between parenting, callous-unemotional traits, and antisocial behavior in youth? A systematic review of evidence. *Clinical Psychology Review*, *33*(4), 593–608. 10.1016/j.cpr.2013.03.001 23583974 10.1016/j.cpr.2013.03.001

[CR69] Waller, R., Hyde, L. W., Klump, K. L., & Burt, S. A. (2018). Parenting is an environmental predictor of callous-unemotional traits and aggression: A monozygotic twin differences study. *Journal of the American Academy of Child & Adolescent Psychiatry*, *57*(12), 955–963. 10.1016/j.jaac.2018.07.882 30522741 10.1016/j.jaac.2018.07.882 PMC6296820

[CR71] Waschbusch, D. A., Baweja, R., Babinski, D. E., Mayes, S. D., & Waxmonsky, J. G. (2020). Irritability and limited prosocial emotions/callous-unemotional traits in elementary-school-age children. *Behavior Therapy*, *51*(2), 223–237. 10.1016/j.beth.2019.06.007 32138934 10.1016/j.beth.2019.06.007

[CR72] Yildirim, B. O., & Derksen, J. J. L. (2015). Clarifying the heterogeneity in psychopathic samples: Towards a new continuum of primary and secondary psychopathy. *Aggression and Violent Behavior*, *24*, 9–41. 10.1016/j.avb.2015.05.001

[CR73] Zych, I., Ttofi, M. M., & Farrington, D. P. (2019). Empathy and callous–unemotional traits in different bullying roles: A systematic review and meta-analysis. *Trauma Violence & Abuse*, *20*(1), 3–21. 10.1177/1524838016683456 10.1177/1524838016683456 30803395

