# Supplementary material for: Parenting and Peer Victimization in the Development of Callous-Unemotional Behaviors: Moderation by Irritability and Basal Cortisol
Source: Res Child Adolesc Psychopathol. 2025 Jun 23;53(9):1311–23. doi: 10.1007/s10802-025-01343-9 (PMC12341471; doi:10.1007/s10802-025-01343-9)
Supplement: Supplementary file 1 — Supplementary Material 1 [file 10802_2025_1343_MOESM1_ESM.docx]

**Correlational Analyses**

Correlational analyses indicated that CU behaviors demonstrated moderate-to-high stability from T1 to T2. Physical and relational victimization were highly correlated, consistent with prior work in this developmental period using questionnaire methods (Garnier & Lemerise, 2007; Ostrov et al., 2008). T1 authoritarian parenting, irritability, and physical victimization were significantly positively correlated with T1, but not T2, CU behaviors. By contrast, T1 cortisol was significantly negatively correlated with T2 CU behaviors. Significantly higher levels of T2 CU behaviors [*t*(170) = 2.75, *p* = .003, Cohen’s *d* = 0.28] and T1 authoritarian parenting [*t*(221) = 1.95, *p* = .03, Cohen’s *d* = 0.32] were reported for boys relative to girls. No other variables significantly differed by gender. To account for these differences, gender was included as a covariate in all models. Finally, cohort differences were examined given potential Covid-19-related impacts. Cohorts differed in levels of T1 authoritarian parenting [*F*(3, 219) = 3.79, *p* = .01, η^2^ = .05], T1 irritability [*F*(3, 247) = 2.89, *p* = .04, η^2^ = .03], T1 relational victimization [*F*(3, 245) = 4.36, *p* = .005, η^2^ = .05] and T1 cortisol [Welch’s *F*(3, 85.43) = 8.86, *p* < .001]. Bonferroni-corrected *post-hoc* comparisons found that Cohort 4 demonstrated significantly lower levels of authoritarian parenting relative to Cohort 1, Cohort 2 demonstrated significantly lower levels of relational victimization relative to Cohort 4, and Cohort 1 demonstrated significantly lower levels of cortisol relative to Cohorts 2 and 3. No specific cohort differences emerged for irritability. Cohort and gender were included as covariates in all models.

**Missing Data Analyses**

Data were also examined for systematic missingness. Missing data was expected given the longitudinal nature of the study, including the transition to a new school context. At T1, teacher report measures had minimal missing data (5% - 6%), with slightly higher rates of missingness for parent reports (15% - 16%). Little’s test suggested that T1 missing questionnaire data was missing completely at random [MCAR; χ^2^(16) = 19.19, *p* = .26]. T1 cortisol data was missing for 27% of participants due to declining to participate, being ineligible due to medication use, or having otherwise unusable cortisol data (e.g., low weight, implausible values). Cortisol missingness significantly differed by cohort [χ^2^(3) = 23.64, *p* < .001, Cramér’s *V* = .30] with Cohort 1 having significantly lower rates of missing data than Cohort 3 (9.5% vs. 44.3%). Participants with missing data did not significantly differ on any other predictor variables, age, gender, or socioeconomic background (SES; measured using a composite of parent occupation, education, and household income). At T2, 34.6% of participants were missing the parent-reported CU behaviors outcome measure. Those without data on this variable did not significantly differ from those with data on any predictor variables of interest, gender, SES, or age. However, cohorts significantly differed in proportions with missing T2 data [χ^2^(3) = 12.09, *p* = .007, Cramér’s *V* = .21], with Cohort 1 demonstrating significantly lower rates of missingness relative to Cohort 3 (20.5% vs. 44.3%). Data were considered missing at random (MAR), and missing data was accounted for using full information maximum likelihood (FIML).

**Supplemental Figures**

**Supplemental Figure 1**

*Conceptual nested path analysis model*

*
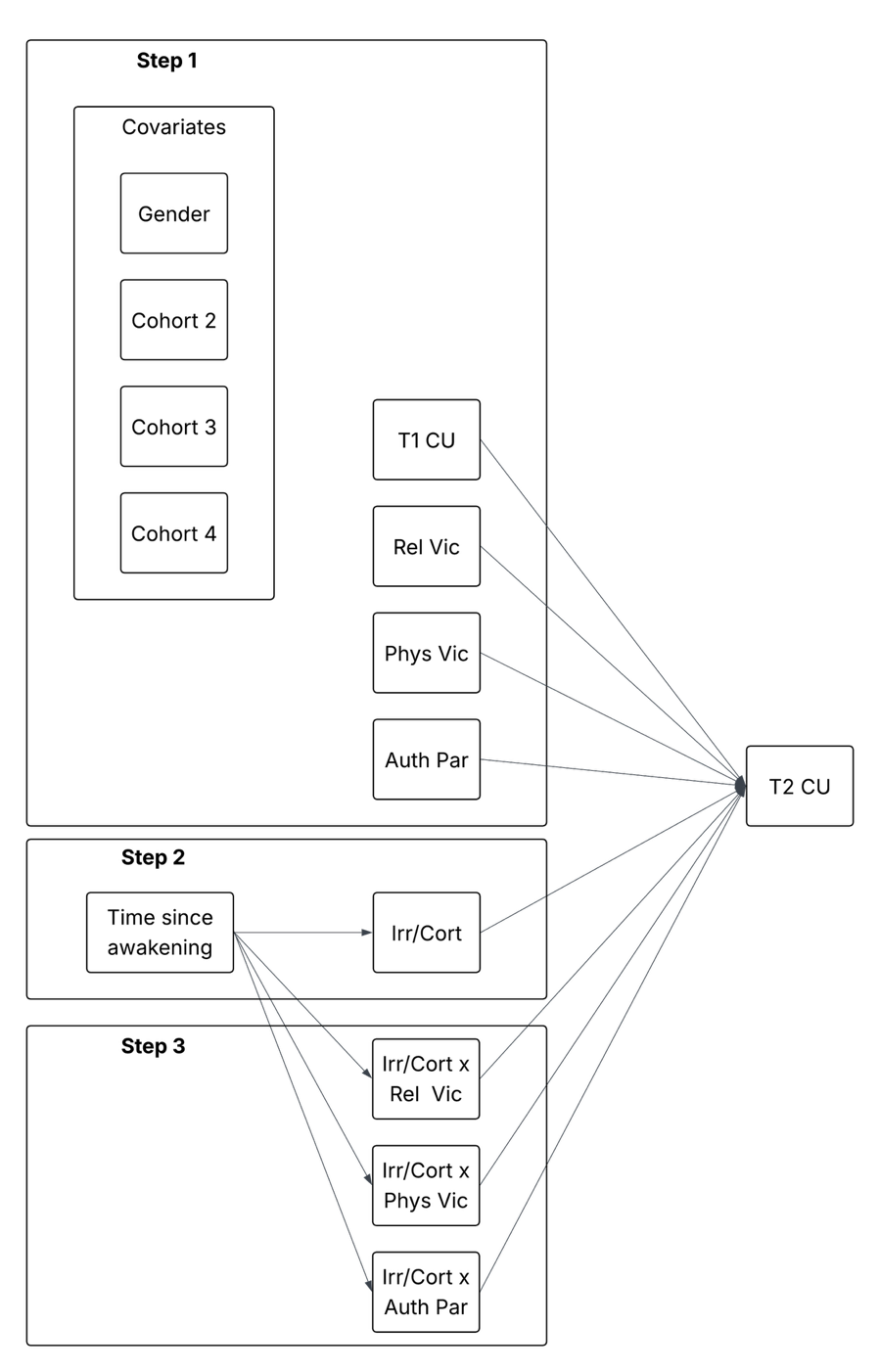
*

*Note.* Irr = irritability, Cort = cortisol, Rel = relational, Phys = physical, Vic = victimization, Auth Par = authoritarian parenting, CU = callous-unemotional behaviors, T1 = time 1, T2 = time 2. All variables were regressed onto exogenous covariates contained within “Covariates” box. Time since awakening was included in cortisol models only. Regression paths to covariates and covariances among variables not depicted for ease of communication. All variables included in “Step 1” were entered, then those in “Step 2” were added, and finally those in “Step 3” were added. Irritability and cortisol models were conducted separately.
